# Immunoprofiling of T cell responses in melanoma patients undergoing CPI therapy

**DOI:** 10.1186/2051-1426-3-S2-P326

**Published:** 2015-11-04

**Authors:** Lee Ann Talarico, Daniel Grubaugh, Zheng Yan, Aula Alami, Jean-Luc Bodmer, Darren E Higgins, F Stephen Hodi, Jessica Flechtner

**Affiliations:** 1 Genocea Biosciences, Inc, Cambridge, MA, USA; 2 Harvard Medical School, Boston, MA, USA; 3 Dana-Farber Cancer Institute, Boston, MA, USA

## 

Successful treatment of melanoma patients with checkpoint inhibitors (CPI) has reinforced the importance of T cells in anti-tumor efficacy. Despite significant progress, CPI therapy is effective in only 40-50% of treated subjects, with substantial toxicity. As a result, it is imperative to understand the profile of T cell responses to tumor antigens, to determine if patterns of responsiveness can be identified for those subjects who respond to immunotherapy. ATLAS^TM^ is a T cell antigen discovery platform in which putative antigens are expressed as individual clones that can be processed by any subject's antigen presenting cells and presented as peptide epitopes in the context of their own MHC class I or II molecules. If autologous CD4^+^ or CD8^+^ T cells are added that are specific for a given clone in a given well, a readout of activation can be measured. We hypothesized that the ATLAS™ technology could be applied to characterize and profile the T cell responses to tumor-associated antigens (TAA) of diverse human subjects undergoing CPI therapy. As a proof of concept, an expression library containing 23 full-length melanoma TAA was constructed and used to interrogate memory CD4^+^ and CD8^+^ T cell responses from of a cohort of melanoma patients who have undergone treatment with pembrolizumab. All 23 TAA were cloned and sequence verified; 96% and 91% were successfully expressed in the CD4-specific and CD8-specific library, respectively. Peripheral blood mononuclear cells (PBMC) were collected from ten patients who had undergone immunotherapy with pembrolizumab; CD4^+^ and CD8^+^ T cells were sorted and non-specifically expanded, and monocytes differentiated into dendritic cells (MDDC) *in vitro*. PBMC yields were comparable to historical assay data with the exception of lower viability of MDDC. Memory T cell responses to TAA were detectable and this study suggests that T cell responses to TAA can be measured in multiple, HLA-diverse subjects during CPI therapy, without the need to derive cell lines or use predictive algorithms. Work is currently underway to increase both the number of antigens and participants evaluated. Analyses will include the frequency and breadth of responses and the relationship between CD4^+^ and CD8^+^ T cell responses in subjects who benefit from CPI therapy compared with those who do not. This work has implications for both patient stratification and identification of novel immunotherapies.

